# Between Waves and Diffusion: Paradoxical Entropy Production in an Exceptional Regime

**DOI:** 10.3390/e20110881

**Published:** 2018-11-16

**Authors:** Karl Heinz Hoffmann, Kathrin Kulmus, Christopher Essex, Janett Prehl

**Affiliations:** 1 Institute of Physics, Chemnitz University of Technology, D-09107 Chemnitz, Germany; 2 Department of Applied Mathematics, University of Western Ontario, Middlesex College, London, ON N6A 5B7, Canada

**Keywords:** entropy production paradox, entropy, fractional diffusion

## Abstract

The entropy production rate is a well established measure for the extent of irreversibility in a process. For irreversible processes, one thus usually expects that the entropy production rate approaches zero in the reversible limit. Fractional diffusion equations provide a fascinating testbed for that intuition in that they build a bridge connecting the fully irreversible diffusion equation with the fully reversible wave equation by a one-parameter family of processes. The entropy production paradox describes the very non-intuitive *increase* of the entropy production rate as that bridge is passed from irreversible diffusion to reversible waves. This paradox has been established for time- and space-fractional diffusion equations on one-dimensional continuous space and for the Shannon, Tsallis and Renyi entropies. After a brief review of the known results, we generalize it to time-fractional diffusion on a finite chain of points described by a fractional master equation.

## 1. Introduction

Physical processes are naturally separated into two broad classes: reversible and irreversible. Time reversal for the former leads to possible physical processes while for the latter implies manifestly unphysical outcomes. Mathematical descriptions representing irreversibility are correspondingly not time reversal invariant: the equations backward in time are different from those evolving forward in time.

Typical examples for irreversible dynamics include diffusion processes either describing molecular or turbulent diffusion as well as heat conduction or chemical reactions. The diffusion equation

(1) $$\begin{array}{c} \frac{\partial }{\partial t}\;P\left(x,t\right)=\frac{\partial^{2}}{\partial x^{2}}\;P\left(x,t\right) \end{array}$$

is the representative example, where P(x,t) is a probability distribution function (PDF) that describes the probability to find a particle at a certain position *x* at time *t*. For the diffusion equation, solutions running backward in time correspond to a process of “undiffusing”: for example, diluted ink, under such a process, would gather itself back into the initial drops of inks added to the mixture.

That is not so for reversible processes. Typical examples for reversible dynamics are Hamiltonian mechanics, quantum mechanics, and classical electrodynamics. These are iconically representable by the wave equation

(2) $$\begin{array}{c} \frac{\partial^{2}}{\partial t^{2}}\;P\left(x,t\right)=\frac{\partial^{2}}{\partial x^{2}}\;P\left(x,t\right), \end{array}$$

which is unchanged under time reversal. All that happens is the reversal of the propagation direction, which remains physical. If a light wave encounters a mirror, it travels just back the way it came. Furthermore, wave phenomena are characterized by propagation, unlike diffusion which is characterized by dispersion without propagation.

These fundamental differences between the diffusion and the wave equation are also apparent on mathematical grounds and in their solutions. While the former has evolutionary relaxation over all space scales to infinity, the latter has finite speed propagation. Mathematically, the diffusion equation is parabolic and has one characteristic, whereas the wave equation is hyperbolic with two characteristic solutions. In addition, the numerical treatments of parabolic versus hyperbolic equations are different too.

As a result, these two prototypical equations seem to be clearly separated and unconnected. It was thus a fascinating undertaking to seek out a way to join these two worlds nonetheless and to study what the consequences are. The first way to accomplish such a connection was to explore the use of fractional calculus in the context of this equation

(3) $$\begin{array}{c} \frac{\partial^{\gamma }}{\partial t^{\gamma }}\;P\left(x,t\right)=\frac{\partial^{2}}{\partial x^{2}}\;P\left(x,t\right). \end{array}$$

The fractional derivative $\frac{\partial^{\gamma }}{\partial t^{\gamma }}\;P\left(x,t\right)$ is actually defined via an integral as

(4) $$\begin{array}{cc} \frac{\partial^{\gamma }}{\partial t^{\gamma }}\;f\left(t\right) & =\frac{d^{n+1-k}}{dt^{n+1-k}}\int_{0}^{t}\frac{{\left(t-\tau \right)}^{-\beta }}{\Gamma (1-\beta )}\frac{d^{k}f\left(\tau \right)}{d\tau^{k}}d\tau , \end{array}$$

with γ=n+β and β∈(0,1). As solving the (fractional) diffusion equation is essentially an initial value problem, Davison and Essex [1] were first to prove that only the k=n+1 case works for normal initial value problems. Thus, the fractional derivative is given as

(5) $$\begin{array}{cc} \frac{\partial^{\gamma }}{\partial t^{\gamma }}\;f\left(t\right) & \overset{\left[k=n+1\right]}{=}\int_{0}^{t}\frac{{\left(t-\tau \right)}^{-\beta }}{\Gamma (1-\beta )}\frac{d^{n+1}f\left(\tau \right)}{d\tau^{n+1}}d\tau , \end{array}$$

which is also known as Caputo derivative [2].

For the time-fractional diffusion Equation (3), the domain is t≥0 and x≥0. Negative *x* adds nothing due to spatial symmetry about the origin. This results in an evolution equation that fully contained both the diffusion, γ=1, and the wave equation, γ=2, as special cases—thus for 1≤γ≤2 represents a bridge between two different worlds [3,4,5,6].

The obvious probe to explore this extraordinary bridging regime is something that assesses the differences in the property of reversibility as one traverses between diffusion and waves. Classical (Shannon) entropy

(6) $$\begin{array}{c} S\left(t\right)=-\int_{0}^{\infty }P\left(x,t\right)\ln P\left(x,t\right)dx, \end{array}$$

and particularly entropy production, is the obvious first consideration as an appropriate measure such that the entropy production rate (EPR) [3,7,8] quantifies how irreversible a process is:

(7) $$\begin{array}{c} \dot{S}\left(t\right)=-\int_{0}^{\infty }\dot{P}\left(x,t\right)\ln P\left(x,t\right)dx. \end{array}$$

The reversibility and irreversibility of the wave equation and the diffusion equation respectively manifests itself in the entropy production occurring during the time evolution. Then, in the diffusion case, one has a positive entropy production while for the wave propagation the entropy production is zero. One thus suspects that, for the transition from the diffusion case γ=1 to the wave case γ=2, the EPR will decrease and become zero in the reversible case. However, this expectation has been shown to be erroneous and the corresponding phenomenon has been dubbed the entropy production paradox for fractional diffusion [3]. The entropy production paradox exhibits remarkable robustness, but that 44 does not mean it is universal [9,10]. A key question is why it exists at all.

In this short note, we will review the original results for the entropy production paradox [3,6] in the time-fractional context that arises from the above equations and why, taking particular note to how the symmetries of the extraordinary differential Equation (3) lead to the unexpected result: the EPR increases as the solution approaches the reversible limit.

Furthermore, we will also sketch out how this scaling symmetry plays out in a formally different extraordinary differential equation with space-fractional derivative, with completely different solutions and domains, leading to the same remarkable outcome [6,11]: the EPR increases in the reversible limit. We will also observe in passing that this paradox persists even for generalizations of entropy available in the literature such as Tsallis and Renyi entropies [4,6,12,13,14]. In this context, we want to note that there might be implications of the entropy production paradox on applications known in finance [15,16], ecology [17], computational neuroscience [18], and physics [19,20,21].

We will then widen the scope of the entropy production paradox by leaving the realm of systems continuous in space and investigate the entropy production for time-fractional diffusion discrete in space. We generalize a classical master equation describing diffusion on a chain of points by replacing the time derivative with a time-fractional one, thus opening the full range up to the wave equation on the chain. We find an EPR rich in features. In particular, the scaling symmetry persists even in this finite discrete scenario governed by a master equation picture allowing the paradoxical behavior to reveal itself until the finite size effects dominate. We can thus conclude that this paradoxical behavior is not only unique but extraordinarily robust.

## 2. The Paradox for Time-Fractional Diffusion

The time-fractional problem (3) is fully realized with the following initial conditions:

(8) $$\begin{array}{ccc} P(x,0) & =\delta (x), & \;\mathrm{for}\;0<\gamma \leq 1, \end{array}$$

(9) $$\begin{array}{ccc} P(x,0) & =\delta \left(x\right),\;\frac{\partial }{\partial t}\;P\left(x,t\right){|}_{t=0}\equiv 0, & \;\mathrm{for}\;1<\gamma \leq 2, \end{array}$$

where δ(x) represents the delta distribution.

The solution of Equation (3) is known in terms of the *H*-function (for details, see [5,22]) as

(10) $$\begin{array}{cc} P(x,t) & =\frac{1}{2\;\sqrt{\pi }t^{\gamma /2}}\;H_{1,2}^{2,0}\left[\left.\frac{x}{2\;t^{\gamma /2}}\right|\begin{array}{c} \left(1-\frac{\gamma }{2},\frac{\gamma }{2}\right)\\ \left(0,\frac{1}{2}\right),\left(\frac{1}{2},\frac{1}{2}\right) \end{array}\right]. \end{array}$$

Note that, for each γ, a different P(x,t) is obtained. In accordance with [23], the second initial condition in Equation (9) is set to be zero to guarantee the continuity at γ=1.

### 2.1. The Transformation Group

Interesting as the *H*-function is in its own right, it has little to do with the nature of the regime properties in terms of entropy production. This can be seen by considering the similarity group $x=\lambda^{a}\tilde{x}$ and $t=\lambda^{b}\tilde{t}$, choosing a/b=γ/2. Equation (3) is invariant under this group and thus solutions, G(η), can be found in terms of a similarity variable,

(11) $$\begin{array}{c} \eta =\frac{x}{t^{\frac{\gamma }{2}}}. \end{array}$$

We note that G(η) is a solution of an auxiliary equation [3], which is not relevant here.

While the probability distributions P(x,t) is not a similarity solution in its own right, the function G(η) with its scaling property is essential to the form of P(x,t), which must have a time independent integral over the domain, Σ, in *x*. With $\Sigma^{\prime }$ being the corresponding domain in η, one finds

(12) $$\begin{array}{cc} 1 & \neq \int_{\Sigma }G\left(\eta \right)dx=t^{\frac{\gamma }{2}}\int_{\Sigma^{\prime }}G\left(\eta \right)d\eta . \end{array}$$

This suggests the primary form for the PDF,

(13) $$\begin{array}{cc} P(x,t) & =t^{-\frac{\gamma }{2}}G\left(\eta \right), \end{array}$$

leading to

(14) $$\begin{array}{cc} 1 & =\int_{\Sigma }P\left(x,t\right)dx=t^{\frac{\gamma }{2}}\int_{\Sigma^{\prime }}t^{-\frac{\gamma }{2}}G\left(\eta \right)d\eta =\int_{\Sigma^{\prime }}G\left(\eta \right)d\eta , \end{array}$$

which confirms the form (13) for the probability density. A quick observation reveals that this is precisely the form of (10).

### 2.2. Entropy Production Directly from the PDF

Inserting Equation (13) into (6) leads to

(15) $$\begin{array}{cc} S(\gamma ,t) & =-t^{-\frac{\gamma }{2}}\int_{\Sigma }G\left(\eta \right)\left(\ln t^{-\frac{\gamma }{2}}+\ln G\left(\eta \right)\right)dx\\ & =-\int_{\Sigma^{\prime }}G\left(\eta \right)\left(\ln t^{-\frac{\gamma }{2}}+\ln G\left(\eta \right)\right)d\eta \\ & =\frac{\gamma }{2}\;\ln t+C\left(\gamma \right), \end{array}$$

where $C\left(\gamma \right)=\int_{\Sigma^{\prime }}G\left(\eta \right)\ln G\left(\eta \right)d\eta$ decreases monotonically with γ [5]. It follows that for γ<2

(16) $$\begin{array}{cc} \dot{S}\left(\gamma ,t\right) & =\frac{\gamma }{2\;t}, \end{array}$$

which shows decreasing entropy production with time, but increasing entropy production with γ, where increasing γ corresponds to the direction of the reversible limit in γ-space. Moreover, it is clear that this property has nothing whatever to do with the form of the PDF in (10).

This is the primary form of the entropy production paradox. Instead of a decreasing $\dot{S}$ while approaching the reversible wave case, i.e., increasing γ towards 2, the entropy production increases up to a final non-continuous jump at γ=2 down to $\dot{S}=0$.

### 2.3. Entropy as Order Function

Perhaps this paradoxical behaviour is misleading. That is, one can consider each γ as representing a separate system with its own intrinsic rates. Maybe one might argue that the EPR does not represent the physically meaningful ordering of states in this domain.

**Figure 1:**
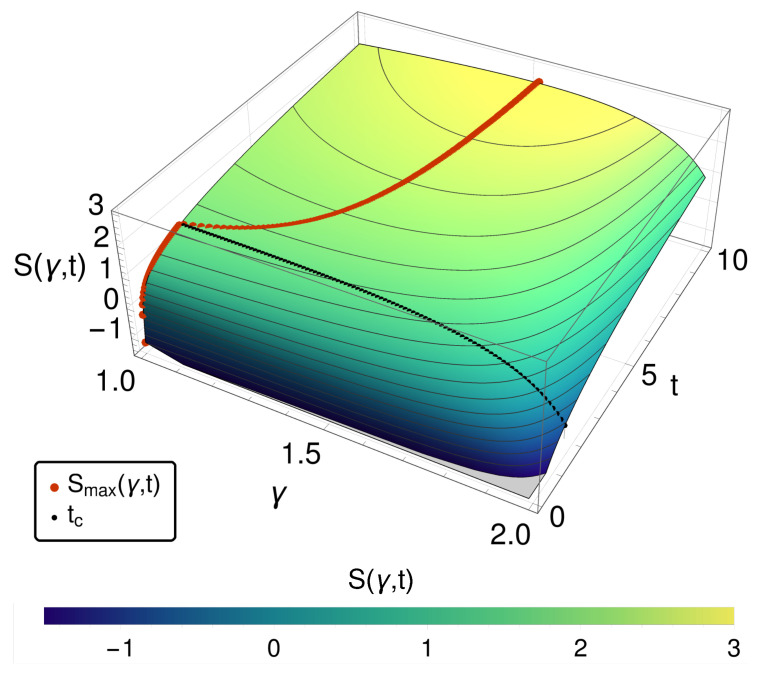
The entropy S(γ,t) is shown over γ and *t*—for small times, a monotonic increasing entropy decreasing γ. At times $t>t_{c}$, a maximum *S* at γ>1 is found for each *t*, as indicated by the red dots.

One alternative is the entropy itself. In Figure 1, the entropy *S* is given over γ and time *t*. For small times, we observe a monotonic decreasing behavior of *S* for increase γ, i.e., approaching towards the reversible case the entropy decreases. However, after a critical time $t_{c}$, a maximum of the entropy within γ∈[1,2) appears. (A detailed discussion can be found in [5].)

Thus, we find that comparing the different probability distributions P(x,t) via the entropy production $\dot{S}$ or via the entropy *S* at constant times does not represent the standard notion of the entropy production or of the entropy that it should decrease towards the reversible case.

This leaves us with entropy production which changes in a paradoxical manner, or entropy which does not even order the states between diffusion and waves because of the maximum. However, perhaps the notion of ordering the respective systems along lines of constant time misses an essential aspect of the matter.

Examination of the evolution of the PDFs computed shows that the solutions between diffusion and waves exhibit properties of both dissipation and propagation simultaneously. The peak of the PDF moves in the half space while the width of the peak broadens. As γ grows, the peak tightens and moves more quickly, until the peak approaches a delta distribution in the γ=2 limit. In this sense, it is a precursor to propagation, which we call *pseudo propagation*, as it is not strict propagation in a mathematical sense.

As this pseudo propagation is more rapid for larger γ, this suggests that the different systems in γ operate with a different internal clocks. Larger γ means the process has greater “quickness”.

For this case, this property may be captured by the rate of movement of the mode of the PDF. Although the mode is not known analytically, it exists and can be determined numerically, whereas for instance the mean or the first moment does not always exist in such problems, as we shall see in the case below.

The time dependent mode for this distribution can be determined from the mode ${\hat{x}}_{\gamma ,t=1}$ at t=1 via

(17) $$\begin{array}{c} \hat{x}=t^{\frac{\gamma }{2}}{\hat{x}}_{\gamma ,1}. \end{array}$$

From this, we can determine a corresponding time, $\tau_{\gamma }$, that puts each system at a similar evolutionary position as a function of γ

(18) $$\begin{array}{cc} \tau_{\gamma }\left(\hat{x}\right) & ={\left(\frac{\hat{x}}{{\hat{x}}_{\gamma ,1}}\right)}^{\frac{2}{\gamma }}. \end{array}$$

**Figure 2:**
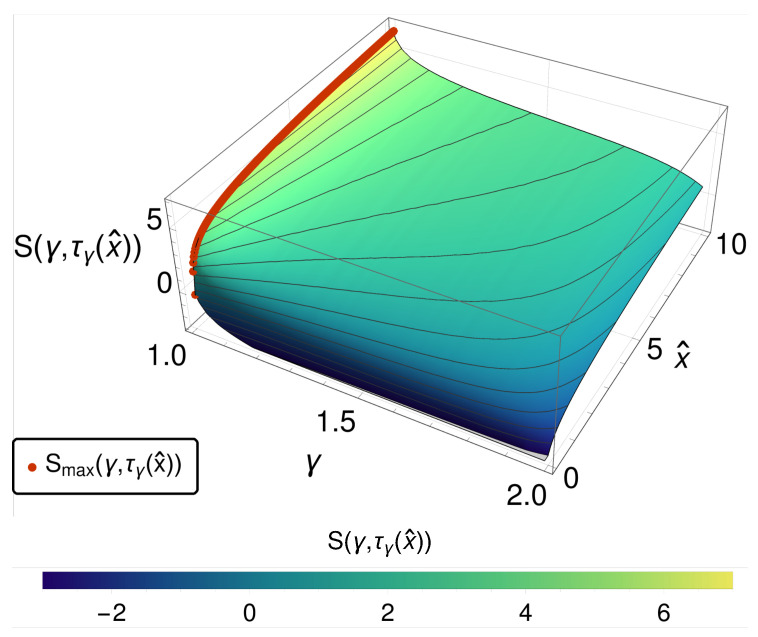
The entropy $S(\gamma ,\tau_{\gamma }\left(\hat{x}\right))$ is shown over γ and $\hat{x}$. We observe a monotonic decreasing function of entropy going from γ=2 to 1. This is emphasized by the red dots, indicating the maximum of *S*, which is always given for γ=1.

Along lines of constant $\hat{x}$ instead of constant *t*, as shown in Figure 2, we observe a monotonic decreasing entropy while crossing from the diffusive to the reversible case. While this does not eliminate the primary paradoxical behavior of the EPR, it causes the entropy itself to operate as an order parameter that has some intuitive content.

## 3. The Paradox Is Not Unique

The entropy production paradox for time-fractional diffusion [3] naturally leads to the question as to whether the paradox is unique. How special and singular is this phenomenon?

It turns out that, while the circumstances that permit this paradox to occur are not universal, they are far from unique. This section provides an alternative circumstance that is as removed from the original paradox as one might get without losing all contact with the context of the problem.

We still must have PDFs that bridge the regime between diffusion and waves with a one-parameter family of PDFs. This can be accomplished by beginning with the diffusion equation and letting the space derivatives decrease in order to 1,

(19) $$\begin{array}{c} \frac{\partial }{\partial t}\;P\left(x,t\right)=\frac{\partial^{\alpha }}{\partial x^{\alpha }}\;P\left(x,t\right), \end{array}$$

where α takes the values (1,2] with α=1 represents the (half) wave equation, while α=2 represents the diffusion equation.

Equation (19) could not be more different for this context than (3). Equation (19) induces an infinite domain, while (3) has a semi-finite one. Here, space does not have an initial point as time does. Time-fractional derivatives imply a nonlocality or “memory” respectively in the their definition [24,25] by the time integration, while space-fractional ones have the classical picture of time in regular differential equations.

A suitable “memory-less” fractional derivative can be found using Fourier transforms. The resulting fractional derivative $\frac{\partial^{\alpha }}{\partial x^{\alpha }}\;P\left(x,t\right)$ is defined via $F\left\{P\right\}=\int_{-\infty }^{\infty }P\left(x\right)\;e^{-ikx}\;dx$ as

(20) $$\begin{array}{c} \frac{\partial^{\alpha }}{\partial x^{\alpha }}\;P\left(x,t\right)=F^{-1}\left\{{\left(i\;k\right)}^{\alpha }F\left\{P(x,t)\right\}\right\}, \end{array}$$

as used in [6,11,12,13], but other definitions are also possible [14]. While the space-fractional derivative is defined through transforms for historical reasons, this does not mean that the time-fractional derivative could not be defined alternatively through a suitable transform.

Utilizing the initial condition P(x,0)=δ(x), the PDF solutions of Equation (19) turn out to be Lévy stable distributions [6,11]

(21) $$\begin{array}{c} P\left(x,t\right)=S\left(\left.x\right|\alpha ,1,{\left(D_{\alpha }\;t\right)}^{\frac{1}{\alpha }},0;1\right) \end{array}$$

with $D_{\alpha }=-\cos\left(\frac{\alpha \pi }{2}\right)$. Note that P(x,t) is defined for −∞<x<∞ and 0≤t<∞.

As in the case of time-fractional diffusion, the solution functions (21) show a scaling behavior in the variables space *x* and time *t* that they can be written as a product of a function of time only and a function of the similarity variable $\eta =x\;t^{\;-\frac{1}{\alpha }}=x\;t^{-\frac{\gamma }{2}}$. Thus, the entropy can then be written in terms of the similarity variable and a scaling function G(η) as

(22) $$\begin{array}{cc} S(\alpha ,t) & =\frac{1}{\alpha }\;\ln t-\int_{\Sigma^{\prime }}G\left(\eta \right)\;\ln G\left(\eta \right)d\eta . \end{array}$$

The analysis of the resulting EPR proceeds in the same fashion as in the time-fractional case and leads again to the entropy production paradox, i.e.,

(23) $$\begin{array}{cc} \dot{S}\left(\alpha ,t\right) & =\frac{1}{\alpha \;t}, \end{array}$$

where here α decreases to the reversible limit meaning the EPR again increases.

**Figure 3:**
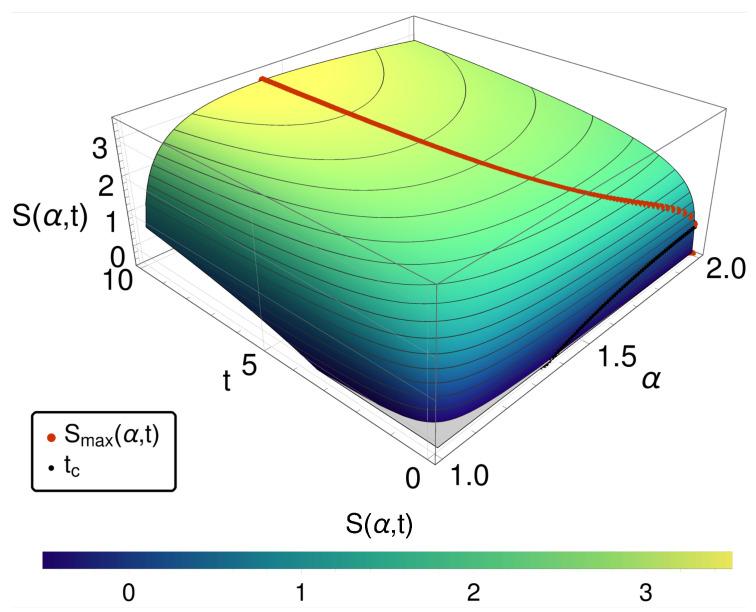
The entropy S(α,t) is shown over α and *t*. For small times $t<t_{c}$, a monotonic increasing entropy for reaching α=2, i.e., the reversible limit, is observed. At larger times, a maximum of *S* at α<2 is found for each *t*, as indicated by the red dots.

**Figure 4:**
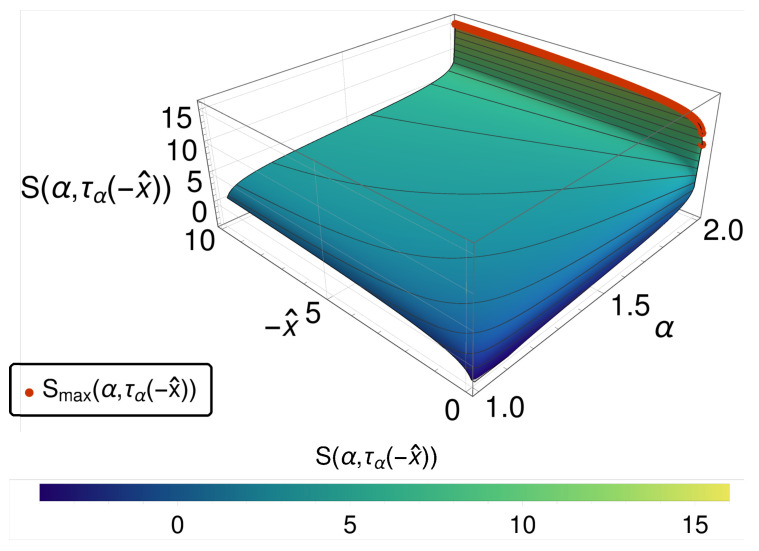
The entropy $S(\alpha ,\tau_{\alpha }\left(-\hat{x}\right))$ is shown over α and $\hat{x}$. We observe a monotonic increasing function of entropy going from α=1 to 2. This is emphasized by the red dots, indicating the maximum of *S*, which is always given for α=2.

As shown in Figure 3, again an entropy maximum in the bridging regime (and thus the paradox) occurs, and the internal clock question recurs. As indicated above, this scenario is one of those, where due to the very different nature of the Lévy stable distributions, i.e., having heavy tails and exhibiting no first moments, only the mode can be used to track the internal clocks of the respective system for each α. In Figure 4, the entropy is given as a function of α and the mode position $\hat{x}$ of the distribution function. It turns out that here again the mode provides an effective strategy to resolve the ordering problem. However, this successful conclusion distracts from the essential difference between the space-fractional and the time-fractional case.

## 4. The Paradox Is Robust

This section makes a small but important point. Does the paradox persist under relaxations in the definitions of entropy itself? It turns out that it does. Entropy generalizations such as Tsallis [26,27,28] and Renyi entropies [8], which include the classical entropy as a limiting case, do not affect the paradox [4,6,11].

In particular, the EPRs for the Tsallis entropy

(24) $$\begin{array}{cc} S_{q}^{T} & =\frac{1}{1-q}\;\int_{\Sigma }P\left(x,t\right)\;\left(1-P^{q-1}\left(x,t\right)\right)\;dx, \end{array}$$

with q∈R\{1} and the Renyi entropy

(25) $$\begin{array}{cc} S_{q}^{R} & =\frac{1}{1-q}\;\ln\left[\int_{\Sigma }P^{q}\left(x,t\right)\;dx\right], \end{array}$$

where q>0, have been analyzed. Both definitions become in the limit of q→1 the classical entropy.

While the extensity parameter *q* makes the resulting EPRs become more complex, both entropies reflecting the paradox demonstrate its robustness.

## 5. Time-Fractional Diffusion on a Finite Interval

The above discussion has already shown that the entropy production paradox occurs in a variety of systems and for a variety of entropy definitions. In this section, we want to show that the entropy production paradox does not only occur in systems using continuous space but also for processes in discrete systems (e.g., [29]).

**Figure 5:**
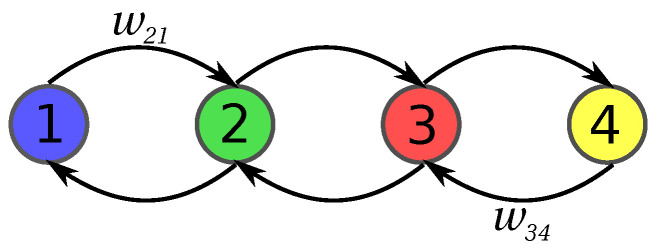
Dynamics on a linear chain of finite length m=4 with only nearest neighbor connections.

We consider the dynamics on a linear chain of finite length *m*. In Figure 5, the setting for a chain with four sites is depicted. The figure visualizes that we treat the case in which only nearest neighbor connections are present. We consider a simple diffusion process on that set with the dynamics of the probability P(i,t) to be in state *i* at time *t* given by the master equation

(26) $$\begin{array}{cc} \frac{d}{dt}\;P\left(i,t\right) & =\sum_{j\neq i}^{m}\left(w_{ij}P\left(j,t\right)-w_{ji}P\left(i,t\right)\right)\\ & =\sum_{i}^{m}w_{ij}P\left(j,t\right), \end{array}$$

where $w_{ij}$ are denoting the transition probabilities from site *j* to site *i*. They are set to $w_{ij}=\kappa$ for |i−j|=1 and $w_{ij}=0$ otherwise, and $w_{ii}=\sum_{j\neq i}w_{ji}$.

We now generalize this master equation by substituting the first order time derivative on the left-hand side of (26) with the fractional derivative as given in Equation (5)

(27) $$\begin{array}{c} \frac{d^{\gamma }}{dt^{\gamma }}\;P\left(t\right)=WP\left(t\right), \end{array}$$

given in matrix-vector notation.

We note that now the $w_{ij}$ no longer represent *transition probabilities* but a connectivity strength indicating how much the value of P(i+1,t) and P(i−1,t) (in addition to P(i,t)) influences the fractional derivative of P(i,t). Below, we will refer to the $w_{ij}$ as generalized transition probabilities.

Equation (27) represents a set of *m* coupled linear time-fractional differential equations. They can be decoupled by an appropriate transition to variables based on the eigensystem of the connectivity strength matrix W with elements $w_{ij}$:

(28) $$\begin{array}{c} We^{\nu }=\lambda_{\nu }e^{\nu }, \end{array}$$

which has *m* eigenvalues $\lambda_{\nu }$, ν=1⋯m and corresponding eigenvectors $e^{\nu }$. In detail, we find

(29) $$\begin{array}{cc} \lambda_{\nu } & =-4\kappa \cos^{2}\left(\frac{\nu \pi }{2m}\right), \end{array}$$

(30) $$\begin{array}{cc} e_{i}^{\nu } & ={\left(-1\right)}^{n+1}\sin\left(\left(2i-1\right)\frac{\nu \pi }{2m}\right). \end{array}$$

The probability distribution can now be expressed as a linear combination of the eigenvectors with coefficients $a_{\nu }\left(t\right)$

(31) $$\begin{array}{c} P_{\gamma }\left(t\right)=\sum_{\nu =1}^{m}a_{\nu }\left(t\right)e^{\nu }. \end{array}$$

Inserting (31) into (27) then leads to a set of *m* decoupled fractional differential equations

(32) $$\begin{array}{c} \frac{d^{\gamma }}{dt^{\gamma }}\;a_{\nu }\left(t\right)=\lambda_{\nu }a_{\nu }\left(t\right), \end{array}$$

which follows from the orthogonality of the eigenvectors.

The resulting solution to this fractional differential equation can be determined for known initial values $a_{\nu }\left(0\right)$ and ${\dot{a}}_{\nu }\left(0\right)$ as

(33) $$\begin{array}{c} a_{\nu }\left(t\right)=a_{\nu }\left(0\right)E_{\gamma }\left(\lambda_{\nu }t^{\gamma }\right)+{\dot{a}}_{\nu }\left(0\right)tE_{\gamma ,2}\left(\lambda_{\nu }t^{\gamma }\right), \end{array}$$

where $E_{\alpha ,\beta }\left(z\right)$ represents the generalized Mittag–Leffler function. It is defined by its power series

(34) $$\begin{array}{c} E_{\alpha ,\beta }\left(z\right)=\sum_{k=0}^{\infty }\frac{z^{k}}{\Gamma (\alpha k+\beta )}\;z,\alpha ,\beta \in C,ℜ\left(\alpha \right)>0,ℜ\left(\beta \right)>0 \end{array}$$

and encompasses the Mittag–Leffler function $E_{\alpha }\left(z\right)=E_{\alpha ,1}\left(z\right)$.

For given $P_{\gamma }\left(0\right)$ and ${\dot{P}}_{\gamma }\left(0\right),$ the corresponding $a_{\nu }\left(0\right)$ and ${\dot{a}}_{\nu }\left(0\right)$ are set by

(35) $$\begin{array}{cc} a_{\nu }\left(0\right) & =\frac{P_{\gamma }\left(0\right)\cdot e^{\nu }}{e^{\nu }\cdot e^{\nu }}, \end{array}$$

(36) $$\begin{array}{cc} {\dot{a}}_{\nu }\left(0\right) & =\frac{{\dot{P}}_{\gamma }\left(0\right)\cdot e^{\nu }}{e^{\nu }\cdot e^{\nu }}. \end{array}$$

Combining the results, we obtain

(37) $$\begin{array}{cc} P_{\gamma }\left(t\right) & =\sum_{\nu =1}^{m}e^{\nu }\left[a_{\nu }\left(0\right)E_{\gamma }\left(\lambda_{\nu }t^{\gamma }\right)+{\dot{a}}_{\nu }\left(0\right)tE_{\gamma ,2}\left(\lambda_{\nu }t^{\gamma }\right)\right] \end{array}$$

as well as its time derivative

(38) $$\begin{array}{cc} {\dot{P}}_{\gamma }\left(t\right)= & \sum_{\nu =1}^{m}e^{\nu }a_{\nu }\left(0\right)\lambda_{\nu }t^{\gamma -1}E_{\gamma ,\gamma }\left(\lambda_{\nu }t^{\gamma }\right)\\ & +\sum_{\nu =1}^{m}e^{\nu }{\dot{a}}_{\nu }\left(0\right)\left[E_{\gamma ,2}\left(\lambda_{\nu }t^{\gamma }\right)+\lambda_{\nu }t^{\gamma }\left(E_{\gamma ,\gamma +1}\left(\lambda_{\nu }t^{\gamma }\right)-E_{\gamma ,\gamma +2}\left(\lambda_{\nu }t^{\gamma }\right)\right)\right]. \end{array}$$

Finally, the entropy production rate is determined

(39) $$\begin{array}{cc} {\dot{S}}_{\gamma }\left(t\right)= & -\sum_{i=1}^{m}{\dot{P}}_{\gamma }\left(i,t\right)\ln P_{\gamma }\left(i,t\right)\\ = & -\sum_{i=1}^{m}\left[\sum_{\nu =1}^{m}e_{i}^{\nu }\left\{a_{\nu }\left(0\right)\lambda_{\nu }t^{\gamma -1}E_{\gamma ,\gamma }\left(\lambda_{\nu }t^{\gamma }\right)\right.\right.\\ & \left.+{\dot{a}}_{\nu }\left(0\right)\left[E_{\gamma ,2}\left(\lambda_{\nu }t^{\gamma }\right)+\lambda_{\nu }t^{\gamma }\left[E_{\gamma ,\gamma +1}\left(\lambda_{\nu }t^{\gamma }\right)-E_{\gamma ,\gamma +2}\left(\lambda_{\nu }t^{\gamma }\right)\right]\right]\right\}\\ & \left.\cdot \ln\left(\sum_{\mu =1}^{m}e_{i}^{\mu }\left[a_{\mu }\left(0\right)E_{\gamma }\left(\lambda_{\mu }t^{\gamma }\right)+{\dot{a}}_{\mu }\left(0\right)tE_{\gamma ,2}\left(\lambda_{\mu }t^{\gamma }\right)\right]\right)\right]. \end{array}$$

**Figure 6:**
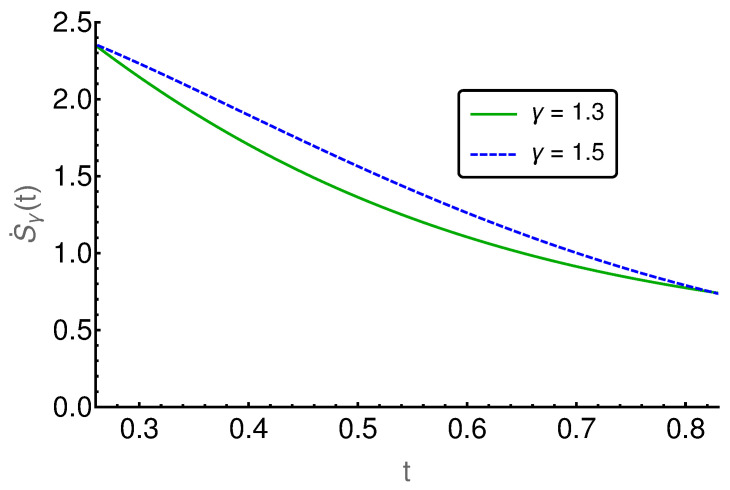
The entropy production rate is given for the short time regime t∈[0.26,0.83]. Here, it behaves paradoxically, as the entropy production rate for γ=1.5 is larger than for the γ=1.3.

**Figure 7:**
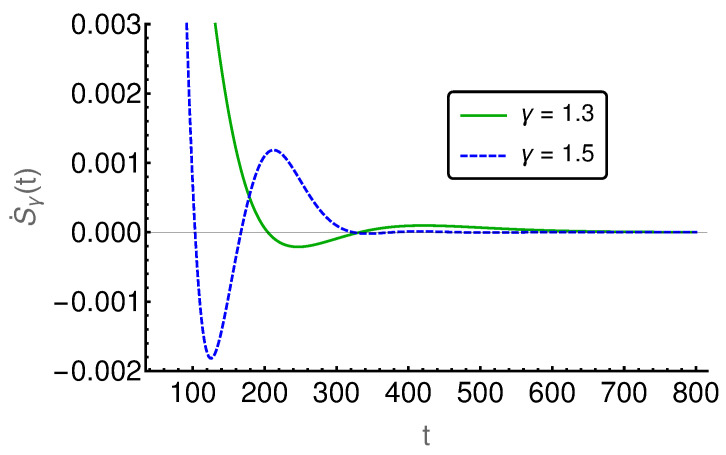
The entropy production rate is given for the intermediate time regime t∈[100,800]. The entropy production rates are crossing each other several times, i.e., the entropy production shows alternating normal and paradoxical behavior.

**Figure 8:**
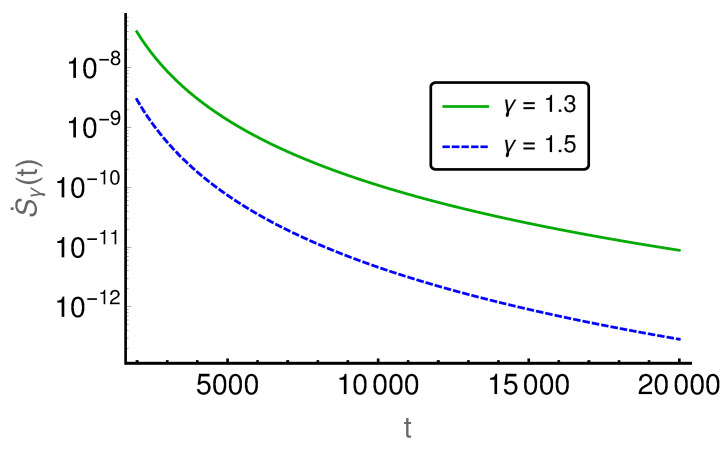
The entropy production rate is given for the long time regime *t*∈[2000, 20,000]. Here, it shows regular ordering with a higher production rate for smaller γ.

**Figure 9:**
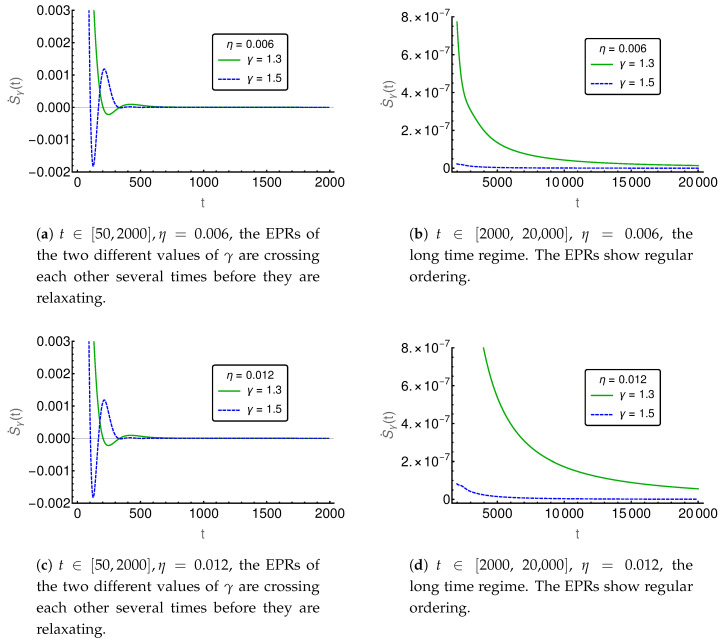
The entropy production rate (EPR) is shown as a function of time for two different values of γ and η.

**Figure 10:**
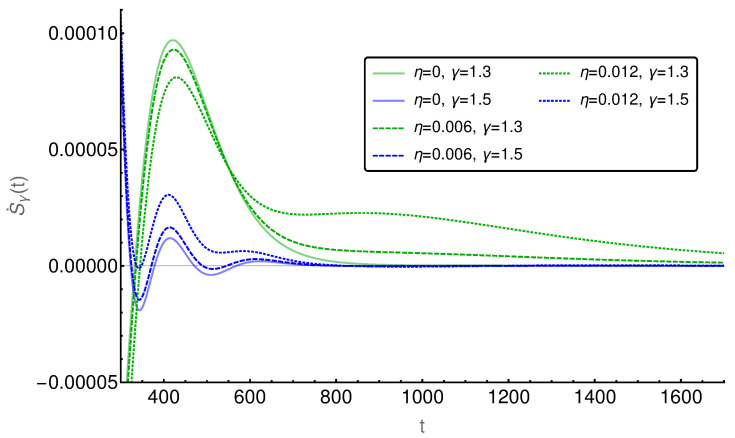
Comparison of the entropy production rate for different η. The influence of the initial probability change on the smaller value of γ persists longer.

In the following figures (Figure 6, Figure 7, Figure 8, Figure 9 and Figure 10), the EPR is shown for m=129 and $P\left(i,0\right)=\delta_{i,(m+1)/2}$ and $\dot{P}\left(i,0\right)=\eta \left(\delta_{i,(m-1)/2}-\delta_{i,(m+3)/2}\right)$, where η indicates the strength of the initial probability change. In Figure 6, Figure 7 and Figure 8, η is set to zero and the EPR is shown for γ=1.3 and γ=1.5. An apparent feature is that the EPRs for different γ cross each other several times. This alone exemplifies that the entropy production paradox is present in certain cases.

If we focus on the small time regime as presented in Figure 6, we can see that the γ=1.5-case has a larger rate than the γ=1.3-case and thus the entropy production paradox is recovered. In the intermediate time regime, between 100 and 800, the two rates cross each other (cf. Figure 7) several times and thus we find the paradox but also the intuitive behavior where the EPR is larger for smaller values of γ indicating larger proximity to the irreversible diffusion process. Finally, for large times, we see in Figure 8 that EPRs show regular ordering in the final relaxation process towards equilibrium.

In Figure 9, the EPR is shown for two different values of η, i.e., η=0.006 and η=0.012. Again, the entropy production paradox appears in the small time regime, while regular behavior is observed for long time periods. Interestingly, the initial probability change η shows only a small influence for short and intermediate times (see Figure 9a,c), as both EPRs apparently behave in the same way as for η=0. For long time periods, as shown in Figure 9b,d, we observe a stronger influence of η=0.012 than of η=0.006 as expected and additionally we find that, for smaller values of γ, the influence of η is stronger than for larger γ.

A further comparison of the EPRs for different initial probability changes η (cf. Figure 10) shows that the influence of η persists longer for γ=1.3 than for γ=1.5. Furthermore, it can be seen that at least for the smaller values of γ the initial probability change does not lead automatically to a larger or lower EPR. Whether the EPR is raised or reduced depends on time. This is an exciting finding, which needs further investigation.

## 6. Conclusions

The first encounter with the entropy production paradox was realized by an *increase* of the entropy production rate (EPR) as the parameter γ, representing the fractional order of the time derivative in the time-fractional diffusion equation, is moved from γ=1, the diffusion case, to γ=2, the wave case. Subsequently, it was realized that this was not unique. Substantively different cases, as the space-fractional diffusion equation, were found where the EPR increased as the control parameter approached the reversible limit. This unexpected behavior contradicts the expectation that the EPR ought to decease as the reversible limit is approached. Furthermore, we have shown that this peculiar behavior is robust, even valid for generalized entropies.

Recently, we extended the analysis to time-fractional diffusion on a finite chain of points. This extension promises new insights as the scaling features present in the fractional diffusion on a half-infinite space no longer exist due to the confinement of the probability in the chain. By a numerical analysis of the EPR, we could establish the existence of the entropy production paradox for short time periods, which are here characterized by the time span in which the distribution initially starting as a δ-distribution in the middle of the chain has not “seen” one of the ends. Thereafter, a complex behavior appears due to the distribution sloshing against the reflecting ends of the chain. The further analysis of this complex behavior is left open here for further research. The treatment of these systems in terms of fractional entropies may be of interest in future work [30,31,32].
